# Minding the gut: extending embodied cognition and perception to the gut complex

**DOI:** 10.3389/fnins.2023.1172783

**Published:** 2024-01-08

**Authors:** Federico Boem, Gregor P. Greslehner, Jan Pieter Konsman, Lynn Chiu

**Affiliations:** ^1^Section Philosophy, University of Twente, Enschede, Netherlands; ^2^Department of Philosophy, University of Vienna, Vienna, Austria; ^3^IMMUNOlogy from CONcepts and ExPeriments to Translation, CNRS UMR, University of Bordeaux, Bordeaux, France; ^4^Department of Evolutionary Biology, University of Vienna, Vienna, Austria

**Keywords:** gut complex, embodied cognition, perception, proto-cognition, affordances

## Abstract

Scientific and philosophical accounts of cognition and perception have traditionally focused on the brain and external sense organs. The extended view of embodied cognition suggests including other parts of the body in these processes. However, one organ has often been overlooked: the gut. Frequently conceptualized as merely a tube for digesting food, there is much more to the gut than meets the eye. Having its own enteric nervous system, sometimes referred to as the “second brain,” the gut is also an immune organ and has a large surface area interacting with gut microbiota. The gut has been shown to play an important role in many physiological processes, and may arguably do so as well in perception and cognition. We argue that proposals of embodied perception and cognition should take into account the role of the “gut complex,” which considers the enteric nervous, endocrine, immune, and microbiota systems as well as gut tissue and mucosal structures. The gut complex is an interface between bodily tissues and the “internalized external environment” of the gut lumen, involved in many aspects of organismic activity beyond food intake. We thus extend current embodiment theories and suggest a more inclusive account of how to “mind the gut” in studying cognitive processes.

## 1 Premise and aim

Etymologically, the term “cognition” derives from the Latin word “cognitio,” in turn from the verb “cognoscere” which stands for “to know *with*” in the relational sense of “to understand,” but also “to recognize,” “to learn,” and even “to discover.” Roughly, cognitive activities historically concerned processes underlying reasoning, memory, problem solving, understanding, and processing language, etc. The scientific study of cognition in the form of the cognitive sciences in the 1950 and 1960s has been qualified (by some) as a revolution away from behaviorism in psychology (Miller, 2003). Two distinctive features of the cognitive sciences are the proposal that hypothetical constructs, for example Chomsky’s control module of universal grammar, play an important role (Hornstein and Chomsky, 1988), and that, following Fodor, some of these constructs are organized in modules that are implemented in the brain (Fodor, 1985). This perspective has gradually been seen as too anthropocentric (non-human animals display, with various differences, cognitive abilities without using the same tools as their human counterparts) and too restricted to certain assumptions that have proven to be unjustified (for example, reasoning and emotions are not strictly separated; Damasio, 2004).

Before proceeding, it is necessary to make a premise about the term “cognition” and its use. It is a critical point, the site of sometimes heated debates, in many fields of research dealing with cognition. For the purpose of this study, our use of the term cognition is to be understood in a “liberal” and “extended” manner. Within the cognitive sciences, the term cognition is often used to refer to all processes (conscious or unconscious) involving the organization of some knowledge content, including perception and reflection on it. This definition is very general and also rather vague, and often does not help to distinguish cognition from other processes. However, Facchin (2023) has recently shown how different research traditions (usually also belonging to different disciplines and methodologies) adopt different (often irreconcilable) definitions of cognition. For instance, following Facchin, those who regard cognition as a process to be studied exclusively within the so-called cognitive agent perspective (e.g., Fodor, 1980) tend to consider the environment as a given to be processed that remains, however, external to cognition proper. In contrast, ecological or extended embodied approaches to cognition (e.g., Clark, 2003; Chemero, 2011) make the environment a constructive element of cognition. Moreover, other areas of research, which deal with cognition as an emergent property (see McClelland, 2010) or a collective feature arising from interactions between multiple subjects (such as the collective cognition of an anthill, see Gelblum et al., 2020), have adopted still different approaches. Another heated debate is that on possible cognitive characteristics of artificial intelligence.^1^

Since there is no space to treat these debates in much detail here, we feel it is appropriate to acknowledge the existence of a more abstract meaning of cognition, which is considered to be “higher-level” and deeply rooted in a rich history of psychological and neurobiological research. However, we do not approve of this term being reserved exclusively for something with a specific and limited scope, as it would negate other important strands of research. For instance, in the community referring to “embodied cognition,” a broader and more integrational view has been proposed, which our contribution wants to extend further by including some processes of the gut complex (which include intestinal epithelia, enteric nervous system, immune and endocrine system and the microbiome).

Therefore, from an idea that saw cognition as the expression of certain structures devoted to it (e.g., the brain and the nervous system) we have moved, not without difficulties that still persist, to a more functional and broader idea of cognition, which includes other cognitive vehicles (i.e., other than the nervous and cerebral structures) and phenomena that were once considered “low” or “simple” (such as the ability of some fungal molds to explore and navigate a complex environment, Nakagaki et al., 2000).

In this paper, we will try to keep as broad an understanding of cognition as possible, also in view of the changes in research that have taken place in this field in recent decades. In particular, in this perspective, we are interested in two crucial aspects. On the one hand, the so-called theories of embodiment, i.e., the idea that the bodily dimension of a subject has its own, foundational cognitive relevance (and is not merely a material container of the mental that would originate exclusively in brain structures). On the other hand, the idea that forms and structures very different from the nervous ones (such as those found in plants, fungi or colonies of bacteria) can present cognitive characteristics. This cognitive dimension has often been referred to as “minimal” or “basal,” as it is considered to be simpler and more basic than human brain activities (Godfrey-Smith, 2016; Lyon et al., 2021).

In fact, as human beings, we tend to assume that cognition mainly concerns activities that we consider “difficult,” such as calculating something or making a logical inference. However, computer science has shown us that we are able to design machines that can perform these tasks much more efficiently than we can. But machines are still a long way from being able to do things that many humans find intuitive to grasp (such as recognizing the significance of a person, understanding an emotion from facial expressions, feeding fabric into a sewing machine). Furthermore, the cognitive abilities of currently existing “less complex” animals or plants are still evolving. Perhaps it is therefore also necessary here to not define as “basic” or “minimal” the cognitive aspects of biological organisms that are very different (and perhaps even structurally simpler) than we humans.

Another preliminary remark concerns the joint discussion of cognition and perception in this paper. The canonical understanding of “cognition” excludes the processes of perception and action, with the latter two seen as inputs and outputs, respectively, to the faculty of cognition. In this model, the triad constituted by perception (input), cognition (the processing of representations) and action (output) is conceived as occurring in this precise order and distinct from each other. Perceptions inform the cognitive unit, which, in turn, processes specific actions in response to them, see (Hurley, 2001; Zipoli Caiani, 2016). We take issue with this model and therefore, in our work, we address cognition and perception together.

Thus, in this paper we present and defend two related but distinct perspectives.

1. The first stipulates that the gut-complex has plays an important role in the perceptual and cognitive characteristics of the subject as a whole, thus in interaction and synergy with the central nervous system and the brain in ways that have emerged during a long common evolutionary history.
2. Secondly, we show how there are evolutionary and ecological reasons to argue that the gut-complex has features attributable to its own forms of proto-cognition and perception that are in interesting ways independent of other structures normally associated with these phenomena.

In our opinion, there are important reasons to consider these two perspectives together and the evidence we present in this paper points in this direction. Cognition can be considered embodied when it depends on characteristics of the body, for example the gut. Furthermore, we believe that the gut complex as part of the body should be viewed as a constraint on and a distributor and regulator of cognitive activities. Accordingly, we surmise (a) that non-neural parts of the gut complex should be part of constitutive components of cognition and (b) that neural parts and non-neural parts of the gut complex interact to bring about cognitive processes (Foglia and Wilson, 2013). We, therefore, think it is original and timely to consider minimal proto-cognitive features for an organ like the gut, which is composed of different cells and tissues.

## 2 Introduction

One of the main intellectual challenges related to cognition is the so-called “mind–body problem,” which often refers to the question of how to reconcile, mental activities with some material correlate or substrate. The mind–body problem arises from attempts to determine the nature of the relationship between the mind (or consciousness) and the physical, material world—that is, to explain how mental states, events or beliefs are linked to physical states, events or processes. Today, much empirical research is focused on the investigation of the neural correlates of mental states, taking a materialist point of view (e.g., Koch et al., 2016). In this sense, the mind–body problem is very often reframed as the “brain–body problem,” with the understanding that the mind corresponds to brain activity (Jaeger, 1978; Schaal, 2005; Crippen, 2017).

In response to this brain-centrism, “4E cognition” (embodied, embedded, enactive, and extended cognition) has sought to go beyond the brain, considered as the cognitive organ *par excellence*. It aims to interpret mental phenomena as grounded in interactions between the brain, other parts of the body and the environment, and in relation to cognitive and perceptive activities (Newen et al., 2018). The 4E solution to the mind–body problem is to treat the mind as distributed across the body and its connections with the world.

One relatively neglected area in the 4E literature, however, is the potential mental role of the gut. The gut is often seen as a digestive, mechanical organ operating “in the dark,” though historically, it has been a long-standing topic of interest in mind–body interactions (see for instance Mathias and Moore, 2018). The “gut-mind” connection is now receiving revived interest due to recent claims regarding the role of the microbiota-gut-brain axis in a range of mental-related disorders and phenomena (e.g., Mayer, 2011; Cryan and Dinan, 2012; Boem et al., 2021). However, the main question in this respect remains whether these factors are causal, constitutive, or enabling with respect to cognition.

In this paper, we argue that the *gut complex*, which comprises the enteric nervous, endocrine, mucosal immune systems and gut microbiota, does not merely influence cognition and perception, but that—within an embodied paradigm of cognition—it also participates in the constitution of them. As indicated above, we believe that the gut complex as part of the body should be viewed as a constraint on and a distributor and regulator of cognitive activities. In addition, we propose that non-neural parts of the body gut complex are to be counted as constitutive components that can bring about cognition and that these, in interaction with neural parts of the gut complex, establish cognitive processes. To support this, we first present a brief examination of embodiment-related accounts, showing their innovation with respect to the traditional cognitivist framework. We then try to argue, on the basis of evidence, that embodiment (classically based on the mechanisms inherent in sensorimotor neurons and related nerve structures) can be extended to the gut complex. In particular, we show how there are important evolutionary reasons to specifically and prominently consider the cognitive dimension of the gut complex. Accordingly, we propose extending the locus of cognition (beyond the canonical ones like the brain) to include the gut complex specifically, focusing both on certain aspects and features of the immune system and analyzing visceral motility. Finally, based on these assumptions, we show how these new sites of cognition may be candidates for genuine and peculiar affordances that may also shed light on the great functional variety of the gut complex itself.

Taking an embodied approach that extends into the gut requires us to revise classical conceptions in philosophy of psychology and neuroscience that are based on the external senses, especially vision (for related critiques of the canonical view of perception, see for instance, Barwich, 2014).

Classical notions of cognition and perception might not directly apply to the gut complex for several reasons. First of all, the dominant classical perspective of cognition is often based on *computer metaphors* and *mental representation*, which may not be well-suited to make sense of the role of the gut. In addition, the sensing of gut contents and conditions is likely to not only involve the nervous systems, but also components of the endocrine and immune systems, which may involve non-computational processes and non-representational content. Specifically, recent evidence seems to show that the enteric nervous system (as such) exhibits sensory properties that are also determined by interaction with the immune system or mediated by environmental factors (both abiotic and biotic) such as the microbiota (Sylvia and Demas, 2018; Schneider et al., 2022). Moreover, although still a partially unexplored subject of investigation, recent evidence on the systems involved in immune-neuromodulation seems to suggest a deep and complex integration that occurs between different sensing apparatuses, both in terms of mechanisms and structures (Yang et al., 2023). An extremely interesting case in this respect is the one involving enteric chemosensory cells (Moran et al., 2021) or the so-called neuropod cells, which are cells of the intestinal epithelium capable not only of constituting a sensor mediated through endocrine signals but also of interfacing directly with the nervous system (see for instance Kaelberer et al., 2020).

At a more theoretical level, this also means that what we generally call sensory capacity is indeed biologically integrated and (for example) “translated” from the immune to the nervous context. However, precisely because of the variety of biological structures that are involved, and given the ecological and embodied aspect of these sensorial activities (see for instance Saborido and Heras-Escribano, 2023), the sensory capacity of the gut also escapes attempts to be reduced to strictly representational frames or modeled through working metaphors (such as the computational one) that have historically arisen to account for a specifically nervous and cerebral accounts of the perceptive dimension.

Furthermore, regarding the concept of perception,^2^ there seems to be important differences between gut perception and the typical external senses. For instance, in the gut, unconscious perception would not be understood as some kind of accessory response to subliminal stimuli, as is the case for vision, but rather as one of the main characteristics of visceral perception that occurs “largely outside of awareness” (Ádám, 1998). Even when visceral perception reaches consciousness, like in hunger, nausea or pain, they are often hard to describe precisely and much harder to share than perceptions of light or sound (Köteles, 2021). Finally, it has been proposed that visceral perception prevails over perception involving the external senses when attention is drawn to cues from inside the body and vice versa (Pennebaker, 1982). V*isceral perceptions* are historically linked to *interoception*, that is the set of sensory perceptual processes *of* the body and *in* the body, capable of generating sensations, moods and thoughts. According to Sherrington (1906), gut contents are sensed by interoceptive processes, but following Craig’s account (Craig, 2002) only the sensing of the gut tissue itself would qualify as interoception. Here, both the sensing of gut contents as an internalized environment and the condition of gut tissue itself are considered interoceptive and relevant for visceral perception.

In fact, these gut-related perceptual forms may imply a different account of cognition. It might mean that it is impossible to talk about perceptions without considering what the one who perceives—the subject—is *like*. In other words, we will not be able to conceive what gut perceptions are like without also considering the corporeality of the cognitive agent. As a result, cognition can no longer be understood as exclusively rooted in the idea of a dipole composed of the subject or cognitive agent, on the one end, processing the external world or its environment, on the other. Such a perspective is currently complemented by the concept of the *holobiont*, which proposes that hosts and residential microbiota—microorganisms living on and in the organism—as part of a larger whole (Gilbert et al., 2012). This may lead to a revision of the relationship between the organism (i.e., subject) and its “environment,” such that the gut can be considered to have enclosed an environment (the gut content), which, through the mouth and anus, communicates with the broader environment. Indeed, the microbiota, by virtue of its symbiotic-ecological relationship, represents not only a component of the biological individual as a functional whole, but also a component of the environment (Bordenstein and Theis, 2015). Thus, cognition and perception refer to each other in a *co-constructive* process (i.e., cognitive processes inform perception and *vice-versa*). In this sense, cognition refers to a complex whole, beyond the classical divisions between mental and physical, somewhere between the idea of an abstract notion of cognition and that of the materiality of the body.

## 3 Embodiment paradigm(s)

As indicated above, the traditional or classical model of cognitive sciences has been dominated by an approach in which the *cognitive agent* can be described as a processual unit, characterized by the ability to identify information and process it. This image, not coincidentally, recalls the process by which a computer is described: the incoming information (input) is processed through some transformation rules that provide the key to describe and sometimes even explain the behavior (output). In other words, the traditional and dominant model therefore establishes cognition as a linear process that goes from *input* to *output* (the input–output model).

This model usually assumes some version of *representationalism*. This term means that the cognitive subject is able to construct an internal representation of the incoming information (i.e., the external environment, the object of perceptible experience) which then serves to determine the actions in relation to this information. In this model, therefore, the triad constituted by *perception (input)*, *cognition (processing of representations)* and *action (output)* is conceived as occurring in this precise order. Perceptions inform the cognitive unit, which, in turn, processes specific actions in response to them (see Hurley, 2001; Zipoli Caiani, 2016).

The organ considered capable of such processing (from perception to action), the *brain*, is therefore viewed as the main *seat of cognition* and the major actor of perception and responses to it. The umbrella of theories that fall under the *embodiment paradigm* is a reaction to this model. According to these alternative accounts, bodily parts, activities, or content can play a decisive role in the formation and processing of cognitive processes (Goldman and De Vignemont, 2009). The embodiment approach’s main line of argument is that characteristics such as reasoning and perception, which are considered by the traditional model as purely attributable to a cognitive subject (in the representational sense), are instead strictly connected and dependent on characteristics of the body and brain.

Within the embodiment theoretical framework, there are many different distinctions and positions. For example, we can distinguish between those that maintain that cognitive processes are still based on representations, albeit realized in the body instead of just in the brain, and those that, instead, do not appeal to representations at all. In other words, adopting an embodied perspective does not necessarily mean giving up the classical approach. It is possible to develop an account of embodied cognition while remaining within a representational and computational frameworks.^3^ For the purposes of the present work, however, the most interesting accounts of embodied cognition are the non-representational ones, those that distribute cognition to or across the body without relying on the concept of representation. With regards to perception, the *ecological theory* of Gibson (1966, 1979) and the *sensorimotor enactive theory of perception* (O’Regan and Noë, 2001; O’Regan, 2011; Zipoli Caiani, 2014) are some of the more dominant theories of embodied perception. However, these accounts are primarily focused on vision, implicitly assuming that principles of visual perception are generally applicable to all sensory modalities. Similar to classical frameworks of perception, main theories of embodiment are still biased toward the peculiarities of vision. While the idea that cognition could influence perception is not shared by all in the field (Firestone and Scholl, 2016), other authors claim explicitly that “[a] cting is perceiving” (Cañal-Bruland et al., 2016) or emphasize “the neural implausibility of the modular mind,” which can be considered as a prerequisite for postulating that cognition, motivation and action are neatly separable from perception (Hackel et al., 2016).

The two main aspects of Gibson’s ecological theory are that perception is a *direct* process (no centralized regulatory activities are needed) and that *action* is its main purpose. Briefly speaking, the ecological character of Gibson’s proposal is that perception is a pure associative relationship between the cognitive agent and the environment, understood as the operative capacity of the agent to relate itself to the environment, by altering it and even being modified by it. Indeed, the central notion in this proposal is that of *affordance*, which in Gibson’s idea stands for everything that the environment (e.g., the object of perception) “offers” or “displays” to the cognitive subject as material for its actions. Obviously this relationship is dynamic such that as the environment and perspectives of the cognitive subject change, affordances also change. This explains the highly non-representational nature of such an account. Here, to perceive means to orient oneself in a dynamic context in order to be able to accomplish something. Movement and action become the cornerstones of perceptual activity. It is therefore no coincidence that, simplifying a bit, the sensorimotor theory of perception (O’Regan and Noë, 2001; O’Regan, 2011) can be understood in the same vein. This account, in addition to the ecological dimension, also adds to the action *a constitutive* aspect. By this, it is meant that perception as such is dependent on the motor constraints imposed by the body. These possibilities of movement and interaction with the surrounding space play a decisive role in determining not only the modalities of perception but also its nature. It follows that this does not happen in a disjoint, segregated, and independent way with respect to corporeality. This is why it is claimed that these phenomena or processes are constitutively linked to each other (see also Zipoli Caiani and Ferretti, 2017). In other words, actions affect perception. Moreover, this implies that the way actual bodies of cognitive agents are shaped determines their perceptual possibilities. Since they influence the types of action that can be performed, and therefore the affordances in an ineluctable way.

## 4 Extending the embodiment traditional framework: *have some guts*

The embodied perspective has shaken the foundational assumptions of cognitive sciences and opened fruitful fields of investigation. However, many such perspectives rely mostly on *external* sensory-motor circuits (especially between vision and action). Yet corporeality does not end with these aspects. It becomes legitimate to ask whether other, especially internal, aspects of the body are therefore linked to the cognitive and perceptive dimension.

On the one hand, historically, so-called *visceral perceptions* have accompanied human experience from ancient times. References to “gut feelings” are nothing new. However, these sensations have historically been considered a “lower,” “more basic” feeling linked to our heritage once defined as “animal.” They remained distant and therefore separated from the “higher” cognitive phenomena associated with the brain, which is believed to be the seat of higher thought and mental activities (Ádám, 1998).

### 4.1 The gut is important in cognition and perception beyond ensuring energy supply

If one admits that the “mind,” for its functioning, depends on the body, then it follows that cognition and perception require energy supply from the body and is already embodied in that sense. Although cognitive neuroscience and the more widespread use of brain imaging techniques have certainly contributed to a brain-centric view of cognition, it is important to bear in mind that these techniques rely on brain tissue consumption of glucose and oxygen and, hence, on bodily metabolism (Raichle, 1997). However, while energy consumption has been used as a proxy to indicate the presence of cognitive processing, cognitive science dismisses the importance of energy use by assuming a distinction between the “vehicle” (which supports, but is not part of cognitive process or content) versus the “content” of cognition (Hurley, 1998).

In this perspective, for example, it is worth to note that studies on appetite have shown how the cognitive dimension of the phenomenon has physiological consequences on the subject’s ability to digest and metabolize nutrients (Power and Schulkin, 2008). Along these lines, it is now well established that circadian rhythms and the sleep cycle, which influence various biological systems and impact on a variety of organismic activities, are also central to the regulation of appetite, including both the stimulation of nutrition and satiety (Scheer et al., 2013). Considering how the sensation of hunger also fluctuates during the day, the sleep–wake cycle is therefore not only related to visual or auditory stimuli, but also influenced by other forms of perception, such as visceral ones. In all these phenomena, including the example of regulation of hunger and thirst, chemical signals from all kinds of different physiological systems are at play (Jourjine, 2017; McKinley et al., 2019). For example, several lines of evidence now indicate that forms of cognition and brain plasticity that stretch beyond functions and circuits typically associated with food intake are influenced by digestive hormones. These include ghrelin, a stomach-produced hormone that is associated with hunger feelings, as well as insulin and leptin, which signal carbohydrate intake and adipose stocks (Ghosh-Swaby et al., 2022). Thus, studying these systems and their activities independently with a preconception of what is or is not part of their functions is a too one-sided perspective. In order to properly regulate these processes, it is clear that these systems have to constantly interact and regulate each other, like in the case of the gut and the brain—the neural and chemical basis of which is well known.

Organismal activities related to the identification, acquisition, processing, and utilization of energy sources, however, are arguably the primary purpose of cognition and perception. Environmental constraints, adaptations and innovations like cooking, along with the associated physiological changes, have shaped fitness and evolution both in terms of cognition and brain characteristics of animals, including humans (Stevenson and Prescott, 2014; Maille and Schradin, 2017). In turn, it has been argued that brain structures enabling spatial navigation, decision-making, and sociality, have probably improved to facilitate acquisition of food (McLean, 2001; Mattson, 2019). Before the evolutionary origin of the brain, nerve nets controlled the acquisition and distribution of food. We thus consider how the gut that comprises a nerve net, has a specific evolutionary history and is in some ways independent (or parallel) to the development of the traditional cognitive centers. Because of that we claim that the gut complex should be seen as central to embodied perception and cognition beyond merely supplying energy to mental processes.

### 4.2 Taking an evolutionary point of view: proto-cognition in the gut

One way to reduce the many possible ways in which the gut complex would be relevant for cognition in mammals is to study evolutionary ancient organisms with less complex ecology and body plans than mammals. Animals with more distributed nervous systems than that of mammals are purposive and flexible agents that arguably, have a basal, proto, or minimal mental or cognitive life. Examples of the cognitive life of brainless invertebrates with distributed nervous systems,^4^ such as jellyfish or sea sponges, have led many to reconsider whether it is appropriate to base our models of the mind on vertebrates and brain-centric animals (Schnell and Clayton, 2021, see also papers in special issue, Regolin and Vallortigara, 2021). It is not a wild idea that organs can be loci of mental life or even consciousness. Sidney Carls-Diamante, for instance, argues that octopus arms are autonomous cognitive structures that exhibit a form of consciousness (Carls-Diamante, 2022), one that perhaps might best be explained by a predictive processing framework (Carls-Diamante, 2021).

From an evolutionary perspective, the gut, with its complex neural network, is sometimes considered the “first brain” (Furness and Stebbing, 2018), but this should be understood as the “first nervous system” given that there is little centralization in the enteric nervous system (ENS). Indeed, the ENS remains similar across phyla, from *Hydra*, the octopus, to humans. If the ENS is one of the main processors and drivers of behavior (understood in a general way as any kind of activity patterns) in brainless animals, the question is whether it retains such roles in animals with a central nervous system (CNS). Evidence shows that not only did the central nervous system evolve later, it also developed rather *independently* from the ENS, with their separate neurons eventually innervating the visceral organs (Furness and Stebbing, 2018). The CNS did not eventually overrule the ENS. From a developmental point of view, the ENS is a largely self-regulating system even though it interacts with the CNS.

It is also important to bear in mind that so-called primordial emotions, such as thirst, hunger for air and food and pain not only give rise to feelings, but also to longer-lasting intentions and motivations (Denton et al., 2009). So, if primordial emotions like hunger and pain, but also, for example associative conditioning learning can be considered to go back quite some way in evolution, it would make sense to clarify the role of the gut complex in cognition, to go back to the first organisms with a gut. Such organisms would include Cnidaria like *Hydra*, with one opening serving as mouth and anus giving access to a body cavity, and the initial bilaterally symmetric organism with a separate mouth and anus connected by a gut system that had a limited capacity to move and no differentiated sensory organs (Evans et al., 2020; Köteles, 2021). Indeed, one could speculate that during the food-abundance times of the Ediacaran, such ancient worm-like organisms with nerve nets around their body cavity would have “needed only to follow chemical “reward gradients” of odors and taste molecules toward the food” (Feinberg and Mallatt, 2016, p. 62).

Our hypothesis is that the evolution of the first nervous systems was related to that of the first gut cavities. Indeed, it is important to remember here that the first coordinated body movements made possible by a nervous system included most probably gut movements in *Hydra*-like organisms (Shimizu et al., 2004). Approaches based on evolution can be interpreted to suggest that we might reassess what could be considered cognition and favor the proposal of minimal or proto-cognition accounts, given evidence that the enteric nervous system precedes the central nervous system in evolutionary terms. One of the main motivations to consider minimal or proto-cognition criteria is that cognition has most likely not suddenly appeared in its full-blown form, but has probably emerged progressively and perhaps several times during evolution. There is a large consensus to agree that habituation and sensitization as forms of cognition are widespread and can be found in bacteria and plants in addition to animals (Moore, 2004; Van Duijn, 2017; Ginsburg and Jablonka, 2021). The first and simplest nervous systems in the form of diffuse nerve nets without much concentration or centralization can be found in Cnidaria, such as sea anemones and *Hydra* (Furness and Stebbing, 2018; Ginsburg and Jablonka, 2021), which are animals with radial body symmetry capable of limited movement and equipped with little harpoons around a single opening giving access to a gut cavity. Nevertheless, and even though not all Cnidaria have been shown to display associative conditioning learning, some clearly do (Cheng, 2021). In addition, *Echinodermata*, including sea cucumbers and star fishes, which are more mobile animals with a pentaradially body symmetry, a mouth and anus connecting a digestive system and the first characteristics of a more centralized nervous system (Mashanov et al., 2009, 2015), display associative learning (Freas and Cheng, 2022). This suggests that the earliest connections of neurons have been sufficient for forms of associative learning. Interestingly, these same organisms have also long been known to host microbiota (Harris, 1993; Bosch, 2012). Therefore, these findings cannot be interpreted to indicate that associative learning depends only on neuronal connections and suggest the causal relevance of the influence of microbiota. It is thus not only the components of the nervous system that should be investigated when tracing the evolution and origins of proto-cognitive capacities.

## 5 Extending the loci of perception and cognition: gut minds, brain minds, and other minds

We propose that a general satiated state of wellbeing is a background “gut feeling” that modulates embodied cognition, for instance, as a background condition for decision-making on top of which other emotion-driven gut feelings inform, guide, or even disturb cognition. This may indeed be a very fine balance and not only be related to satiety. On the one hand, cognitive vigilance is well-known to be reduced after the consumption of a meal (the “post-lunch dip”; Smith and Miles, 1986), while arousal is increased during acute food deprivation (Chan et al., 2006). On the other hand, it is also important to bear in mind that in a cohort of young men who were semi-starved for half a year, cognitive performance was not found to be altered even though the time needed to perform the cognitive tests was longer (Keys et al., 1950). Self-rated hunger in healthy volunteers was nevertheless found to be correlated with certain aspects of moral decision making (Brown et al., 2020).

It is important to specify here that we consider the gut more as a functionally organized, rather than a purely anatomical, unit and propose to employ the term “gut complex” to emphasize this. As such, it contains important parts of the endocrine, immune and nervous systems and hosts a rich microbiota community. The operations of these systems in the gut, for example the enteric nervous system, are each relatively autonomous from their respective systemic counterparts and contribute to several local compartments and physiological units. Functioning of the gut complex requires components of all four systems—the gut nervous, immune, endocrine, and microbiota systems—to work together to serve as an interface with the outside world, processing food, expelling toxins, and managing trillions of residential bacteria, viruses, protists, fungi, and sometimes, helminths. In addition to these functional roles, the gut complex has also been proposed to be involved in sensory processing, (social) behavior, emotions, general motivational state and attitudes as well as cognition, etc. (Mayer, 2011).

Based on a more extended notion of a gut as a gut complex, we would like to propose that studies on gut microbiota and cognition should consider visceral perceptions as potential mediators of observed effects. Interestingly, the reduced attention after consumption of a meal high in saturated fat is linked to increased circulating concentrations of proteins binding bacterial fragments (Madison et al., 2020). Moreover, over the past decade, numerous claims have been made linking particular composition of gut microbiota to improved measures of cognition. Even though most of the initial claims have been based on work in animals, several trials in humans have recently been done or are in progress. While it is too early to come to a definitive conclusion with certain studies showing beneficial effects (Baldi et al., 2021) and others none (Kelly et al., 2017), we would like to consider what processes could mediate such effects. In this context, it is intriguing to observe that while many studies mention mechanisms involving energy substrates or neurotransmitter precursors, very few, if any, invoke visceral interoception.

That the gut is related to decision-making should not come as a surprise. Philosophers such as Heidegger and Merleau-Ponty have long emphasized that the body is a prerequisite for awareness of ourselves, others and the world (Gallagher and Zahavi, 2013). In Antonio Damasio’s somatic marker hypothesis, visceral feedback is essential to decision making-associated cognition (Damasio, 2004), but has been interpreted in many different ways by various scientists, in part due to the lack of computational models and testable hypotheses (Bartol and Linquist, 2015). Promisingly, the idea that the brain functions as a hypotheses-generator in active inference or predictive coding accounts (Bayes-inspired neuroscience) has seemed to spark renewed interest in the role of visceral sensation in cognition (Allen and Tsakiris, 2018).

To make sense of the background role of gut feelings for cognitive and perceptual activities, we propose to consider the gut complex in the context of an affordance- and predictive coding-based account of cognition and in the framework of minimal or proto-cognition.

### 5.1 The “mental” architecture of the gut

Taking these previously discussed aspects and perspectives seriously, especially when thinking about the evolutionary past of bodily systems, invites us to consider in which other activities the gut complex might be involved in (those that we would traditionally “outsource” to other parts of the body). In this section, we want to entertain the possibility that the gut complex may be involved in and contribute to perception and cognition. This is something that might have been even more prominent earlier in evolution than in modern/higher organisms.

We approach this by considering gut motility as a basis of proto-cognition and by investigating the gut as the main location where the immune system and the microbiota are in contact.

#### 5.1.1 Gut motility as a basis of proto-cognition

As indicated above, the gut can be considered a metaphorical “first brain,” the first coordinated and integrated sensory-motor system of the metazoans. Just focusing on the (enteric) nervous system alone, the gut is one of the few organs in the body with its own autonomous nervous system and the only internal organ with its own sensory neurons (other visceral organs relay sensory input to the CNS) (Spencer and Hongzhen, 2020). Furness and Stebbing (2018) argue that the ENS originated independently from the CNS, evolutionarily and developmentally. It retains its relative autonomy from the CNS, both anatomically and functionally. Keijzer et al. (2013) have proposed that early nervous systems evolutionarily originated as a controller of whole-organism motility through the coordination of contractile and excitable epithelial surfaces (the “skin-brain thesis”). The gut epithelium, which coordinates gut motility (such as peristaltic contractions), is one such surface (Jékely et al., 2015). This “skin-brain thesis” offers a way to ground a fundamental notion of embodied basal cognition (Keijzer and Arnellos, 2017). Going beyond the ENS, the gut endocrine, microbiota and immune systems also contribute to the coordination of gut motility (Rhee et al., 2009; Muller et al., 2014; Kitazawa and Kaiya, 2019). Incorporating these other components of the gut complex, Furness et al. (2013) argue that the entirety of endocrine–neuro–immune–organ-defense interactions constitute a *bona fide* “sensory organ” that collects and generates integrated responses to the world, as presented in the gut lumen.

A new, emergent level of organization may lie in the gut complex. Efforts to include such levels are being made within the field of neuro-immune-endocrine interactions. It is becoming increasingly clear that the enteric neuro, endocrine and immune system are integrated evolutionarily, developmentally, and in terms of cellular and molecular organization (Margolis et al., 2016). Neural-immune cell units such as enteroendocrine cells, for instance, are crucial backbones of the enteric system (Huh and Veiga-Fernandes, 2020; Jakob et al., 2020; Wang et al., 2022). Different types of integrative architecture frameworks have been proposed. For instance, some propose the need to reconceptualize the presence of discrete neuro-immune units that constitute “elements” of gut tissue physiology (Chesné et al., 2019; Godinho-Silva et al., 2019). Others are working on clarifying the concept of neuroimmune integrative circuits (Huh and Veiga-Fernandes, 2020; Jakob et al., 2020). An integrative architecture that includes the gut microbiota and metabolism seems to be the current frontier (Deshpande et al., 2021; Jacobson et al., 2021).

It is not enough, however, to posit that the gut complex is a source of proto, basal, or minimal cognition merely on the basis of its relative autonomy and sensory-motor properties. We propose to take into account the capacities of basal or minimal biologically-embedded cognition proposed by Pamela Lyon that include capacities to sense, assign value to, and integrate incoming signals, that to adapt to the environment, those to learn, retain and anticipate, and that to interact with other entities or parts of a system (Lyon et al., 2021). It is well-known that an isolated segment of the intestine can engage in complex motility without CNS input (the “peristaltic reflex” for historical review; Spencer and Hongzhen, 2020). From this evidence can emerge the commonly held view of the gut complex is that of a set of autonomous reflexes. If this were true, then it should not display adaptivity and flexibility to adjust itself to environmental perturbations. Indeed, it is often assumed, on this view, that flexibility in gut responses involves top-down influence from the brain.

Yet even the most basic operations of the gut—gastrointestinal motility—is not merely a matter of reflex arcs in the enteric nervous system. In fact, gut microbiota can modulate intestinal motility (Rhee et al., 2009). In addition, neuro-immune interactions between enteric neurons and muscularis macrophages fine-tunes gut movement. Gut motility is not a hard-wired reflex, but the result of plastic and flexible crosstalk between muscularis macrophages (a part of the immune system; De Schepper et al., 2018), enteric neurons, and gut microbiota (Muller et al., 2014; Verheijden and Boeckxstaens, 2018). Furthermore, Keijzer et al. (2013) argue that the reflex arc is not at all primitive—instead, following (Pantin, 1956), they consider that reflex arc is a secondary architecture of spinal organisms that simplifies an initially complex diffusive nervous system. It is important to point out here that much simpler systems than diffuse nerve nets, such as bacteria and slime molds, can display adaptive habituation and sensitization as forms of learning (Schemann et al., 2020). In addition, the diffuse nerve nets found in sea anemones (Cnidaria) seem to enable classical conditioning associative learning (Cheng, 2021). Therefore, the gut complex, even with input from the brain, is likely to have some ability to reflexively adapt to (and even anticipate) its external environment. Thus, among some important outstanding questions are those relating to what the gut complex can do on its own and when and how it interacts with the CNS (for example, is the CNS necessary for conditioning of effects that involve the gut complex).

Finally, another case for the gut complex as a perceptual system (beyond a mere sensory system) can be made by appealing to the enactive approach to perception. The gut does not seem to be able to sense its content or environment without movement, as illustrated by disorders related to gut paralysis (Spencer and Hongzhen, 2020). In conclusion, a minimal or proto-cognition view of the gut complex falls into the broad family of embodied cognition accounts as it concerns an important part of the animal body plan, it is both biologically and environmentally embedded (the gut lumen constituting an internalized environment) and can be considered enactive.

#### 5.1.2 The cognitive features of the immune system

The immune system and the brain have traditionally been viewed as two separate physiological systems. Indeed, on the one hand, the immune system’s sole purpose was long considered to be defense against pathogens. The brain, on the other hand, had the status of being immune privileged, a status that was often linked to the understanding that the brain was tightly sealed off by the blood–brain barrier that would allow no access to immune cells or mediators. When interactions between the brain and the immune system were found, in particular with the recognition of immunocompetent resident cells and the possibilities of immune cell infiltration, these are usually framed as “guardians of the brain”—even today (Kwon, 2022). By adopting this framework (and frequent war metaphors), it is difficult to appreciate the rich interactions between the nervous system, immune system, gut, and other physiological systems that do exist and to seriously consider the roles they could play. Instead, it would be worthwhile to try and adopt a more neuro-immune-endocrine-microbiota co-construction-based view (Greslehner et al., 2023).

Cognitive thinking is not new to immunology (Tauber, 1997), especially in attempting to understand what it detects and responds to. In addition, there are more general aspects about immunoperception and cognition (Bhat et al., 2021). These approaches, while definitely welcome in that they connect perceptive and cognitive aspects with the immune system, are limited in at least two ways. Firstly, they adopt a narrow view of the immune system being all about defense and fighting disease, and thus all immune processes centering around it (Rankin and Artis, 2018; Pradeu, 2020). Secondly, they are limited by assuming that anything resembling cognition and perception has to be at least partially representational, sorting things into categories (like “self/non-self”). While there definitely is some merit to thinking along these lines and the questions it raises, however, they also might be too much of a burden of deadlock debates. Immune cognition and perception could be conceptualized much more general and simple: what is required is being able to pick up and respond to signals, and having some internal rule of what to do, given a certain signal. The intricacies of these signaling systems and their general properties are a crucial part of physiological explanations in many different fields, e.g., the role of hormones in microbial endocrinology (Neuman et al., 2015). On a more general level, one could think of the immune system as a cognitive or information-processing system, at least in the sense that chemical and neural signals work together to coordinate and integrate physiological processes. One may call this “representational” to some extent, but some of the trench fights surrounding this issue seem distracting and misleading. How successful a physiological system is, is not at all related to how accurately it can represent, but rather to picking appropriate responses to situations more often than not. The most successful immune system would then not be the one capable of the best possible and most accurate representation, but the one being able to optimally respond to sudden changes (Pradeu et al., 2013; Pradeu and Vivier, 2016). Perhaps there is some kind of categorization (without representation) involved, but the categories of “self/nonself” or “dangerous/nondangerous” are not the appropriate ones. However; Matzinger’s “danger theory” is a welcome shift in perspective, as she explicitly acknowledges signals from other physiological systems (Matzinger, 1994). It is important to consider that trying to stick labels onto these categorizations may already be too anthropomorphic. Indeed, seen as a sender-receiver system updating its distributions of actions in response to certain signals, there may not be a need for any categories or labels at all.

“Every living being categorizes. Even the amoeba categorizes the things it encounters into food or nonfood, what it moves toward or moves away from. The amoeba cannot choose whether to categorize; it just does. […] How animals categorize depends upon their sensing apparatus and their ability to move themselves and manipulate objects. […] Whenever a neural ensemble provides the same output with different inputs, there is neural categorization” (Lakoff and Johnson, 1999, pp. 17–18).

Whether or not we want to ascribe cognitive capacities to the immune system, there can be no do doubt that cognition and immune responses are intimately connected and influence in each other’s functioning (Dantzer, 2018). The connection here might be readily illustrated by the case of autoimmune diseases impairing cognitive abilities (Lim et al., 2013; Griffith et al., 2021). The relevance and shared chemical signals of these systems, however, is not limited to pathology. The more novel and interesting approach is to consider how functioning of the gut, which contains its own immune and nervous systems, can be understood utilizing cognitive concepts usually not ascribed to it.

### 5.2 Relevant accounts for cognitive gut complex functions

In delving into the cognitive dimension of the gut-complex, it is also necessary to show how certain features of the gut-complex can be seen and explained from an extended cognitive perspective that is free from certain traditional approaches.

On the one hand, it is important to remember how perceptual phenomena are not necessarily anchored and reducible to types of sensory experience of human beings, for example vision. Biological structures and systems such as the immune system, for example, not only contribute to the functionality of the organism’s nervous structures, but they themselves possess “sense” systems (often of a biochemical nature) that determine their relationship with the surrounding environment, in terms of anticipation and reaction capabilities.

On the other hand, it is essential to recognize that the motility of the gut-complex is not only directed by higher structures, exclusively devoted to processes such as the digestive one. Although “internal” and hidden from our external human perception, the gut-complex can provide a window to our organism and in relation with our external peripersonal environment.

The term *cognition* can cover many different phenomena and needs to be specified. Human cognition does likely not differ fundamentally from cognition of other evolutionary-closely related animals, but can be assessed more readily by verbal reports. Animal cognitive abilities have been proposed to range from perceiving and sensing to understanding and conceiving of notions (McLean, 2001), but sometimes also refer to more complex phenomena as signs of awareness, and of insight and mental states (Call and Tomasello, 2008). Alternatively, cognition can be broadly considered as neural processing of information from the environment, which then includes acquisition, integration, storage, retrieval of information and decision making (Shettleworth, 2001). Even though the latter perspective seems to limit cognition to organisms with neural tissues, it can be adapted to be applied to organisms without nervous systems, for example in accounts of minimal or proto-cognition. In addition, within the broad context of embodied cognition, it would be important to better understand how environmentally-induced physiological changes can affect cognition (Maille and Schradin, 2017), which is a perspective that is compatible not only with Damasio’s somatic marker hypothesis, but also with an affordance and predictive coding-based account of cognition.

The idea that affordances are action opportunities that the environment offers to an organism is a welcome alternative to classical views of perception, but it has also mostly focused on the external senses, particularly on vision (Withagen and Chemero, 2009; Gibson, 2014).

As mentioned, how perception of an action on the environment is, in part, determined by bodily internal states does not seem to be further developed by Gibson. More recently, however, it has been proposed, in the context of the hierarchical affordance competition hypothesis, that: “Once a given action is selected, it is executed through continuous feedback control, using sensory information from the environment as well as internal predictions of expected feedback to fine-tune and update the ongoing action until completion” (Pezzulo and Cisek, 2016, p. 415). This formulation links affordance-based ecological psychology to internal processes, but also raises the question of what is meant by “internal prediction.”

Damasio’s somatic marker hypothesis of cognition seems compatible with the ways in which active inference or predictive coding models of cognition present interoception and gut (Tsakiris and Critchley, 2016; Pezzulo et al., 2018). Predictive processing or coding is “a general computational principle which can be applied to describe perception, action, cognition, and their relationships in a single, conceptually unified manner” that incorporates top-down and hierarchical processing enabling statistical estimation and error prediction minimization (Wiese and Metzinger, 2017). As such, predictive coding can provide computational approaches that both the somatic marker hypothesis and affordance-based ecological psychology lack.

Interestingly, active inference or predictive coding accounts first emerged as a model of visual processing (Rao and Ballard, 1999). In this context, the brain “represents” the external environment based on the effects that the environment produces on a sensory organ like the eye. An advantageous strategy would be for the brain to anticipate or predict in a top-down manner the sensory input and to only process in a bottom-up way that input from the sensory organ that is different from the prediction. The essential feature to explain by predictive coding mechanisms is the reduction of free energy, understood as an information theory measure (Friston, 2010), rather than a (bio) physical notion.

More recently, interoceptive inference has been presented as an expression of a broader move from homeostasis to allostasis stressing anticipation and prediction more. Applying this framework to interoception has given rise to the Embodied Interoceptive Coding model (EPIC) (Barrett and Simmons, 2015) according to which predictive models “underpin [ning] cognitive representation are entirely subservient to the efficient satisfaction of the body’s physiological requirements” (Corcoran and Hohwy, 2018, p. 282). In this model, the so-called visceromotor brain regions (the cingulate, the posterior ventral medial prefrontal and orbitofrontal cortices, and the ventral portions of the anterior insula) are thought to send predictions to the hypothalamus and brainstem, while the mid and posterior insula “calculates” the error between expected and actual input from the body (Barrett and Simmons, 2015). One level of anticipation/prediction of biological systems is reflected in the fact that many of these systems display cyclic responses that seem follow some biological clock. While studies of biological clocks have in part brain-centered focusing on the suprachiasmatic nucleus of the hypothalamus (Schulkin and Sterling, 2019), it has become clear that many systems including the gut and contained microbiota drive cyclic responses that are in part independent from the “brain clock” (Choi et al., 2021; Segers and Depoortere, 2021).

Over the past years, several authors have indeed considered hunger, satiety, sickness and chronic pain in the context of predictive coding based accounts (Hechler et al., 2016; Crutchfield et al., 2018; Henningsen et al., 2018; Lasselin et al., 2018; Livneh and Andermann, 2021; Tschantz et al., 2022). Although this is hardly surprising for a notion that gained so much traction both in neuroscience and psychology, some recurrent criticisms of predictive coding accounts have also emerged. These concern the (im) plausibility of the widespread biological implementation requirements allowing for predictions, the inherently rewarding aspects of exploring and play, which are full of surprise and the error of supposing that model features correspond to some biological entities (Clark, 2013; Köteles, 2021; Bruineberg et al., 2022).

## 6 Conclusion and outlook

The issues of cognition and perception are receiving ample attention, but often remain stuck in certain views of which structures and functions are involved and doing what (a division of labor we have criticized elsewhere as the “building block model”; Greslehner et al., 2023). This often means that cognition and perception are primarily attributed to the central nervous system or remain rather vague and metaphorical when it comes to other physiological systems—each with their traditionally assigned role. We are convinced there exist more general approaches that are needed to better understand these issues. While efforts are being made to more broadly consider cognitive elements, e.g., of the immune system, such approaches also need to be more general, perhaps building on the principles of predictive processing and coding, which are currently receiving a lot of attention. Triggering an (important) debate about the aspects of representation (if and to what extent an embodied system has to be representational when it comes to cognition and perception). By taking cognition and perception out of their classical “locus” (i.e., brain-centered perspective), a more general view can and needs to be developed.

Many general aspects in these debates are still waiting to be applied to other systems like the gut, but would allow to further develop the concepts of embodied cognition and perception. The role of the immune system and microbiota are often neglected in this debate, which is traditionally centered on the nervous system and muscular aspects. Rather than frontally criticizing existing accounts, we attempt to take things further, toward more general aspects and lessons for scientists and philosophers. Thinking along the lines in which only certain parts/structures of the body are involved in perception/cognition and assigning those parts their traditional functions, including the gut and its broad physiological connections allows breaking out of established schemes for that traditional mapping. The gut is much more versatile, beyond the classically ascribed roles. Brain and body are not separate, the locus of cognition and perception is not brain-centered, and certainly not floating like “brains in a vat.” In addition to scientific discoveries, we are convinced that philosophical contributions are essential in putting these different systems and how they interact in perspective (Laplane et al., 2019).

Categorization can become limiting or biased: e.g. that the gut would only deal with digestion and nutrition, the immune system only with pathogens or diseases, the brain only with cognition—very much like building blocks. That is why the gut is such a good example to break this bad habit. It can be a seat of cognition and perception, and in many ways different than one might have thought. All the better, as more general aspects of philosophy can contribute and be worked out here.

Embodied cognition offers a rich conceptual ground for developing theories about how cognition and perception work, and how different parts of our bodies play a role in these processes. Both notions of *embodiment* and *cognition*, however, have been limited by certain perspectives which neglected an important part of the body, namely the gut. In this paper, we offer an enactive, embodied, and affordance-construction approach to the gut complex. In doing so, we highlight its important role beyond just energy supply, digestion, and other functions usually attributed to the gut. By taking into account scientific evidence and conceptual rethinking, the influence of the gut on the mind is much more interwoven in other basic activities, development, regulation, and overall integrated collaboration of different systems of the body and the mind. We discussed how affordances-, predictive coding-based and minimal or proto-cognition accounts are relevant for pursuing these lines of research in the future.

For these reasons, we have argued that the particular (also in the sense of non-substitutable) contribution of the gut complex to the cognitive and perceptual functions of the organism is to be seen as closely related to the specific perceptual properties of the gut complex as such. In other words, if we recognize that these two perspectives have a theoretical autonomy, we believe instead that the proto-cognitive dimension of the gut complex allows us to extend and broaden the nature of perceptual stimuli and activities in such a way as to make their incorporation into a general account of the cognitive subject’s perception and cognition more coherent.

Making this shift in perspective might “take some guts,” but we are convinced that minding the gut is an important step in overcoming certain gaps that exist in our understanding of how embodied cognition can be further developed in light of the role of the microbiota-gut-brain-immune axis.

Taken together, addressing these and additional issues open up again the study of gut perception.

## Data availability statement

The original contributions presented in the study are included in the article/supplementary material, further inquiries can be directed to the corresponding author.

## Author contributions

All authors listed have made a substantial, direct, and intellectual contribution to the work and approved it for publication.

## Footnotes

^1^Regarding AI and cognition, there is a very wide debate that we think should not be opened (like Pandora’s box) in our article. However, we would like to clarify a few points to avoid misunderstandings. Simplifying, our position is that if “embodied” is taken seriously in embodied cognition, the gut-complex should be included in these considerations. Therefore, an AI cannot have cognition *per se*, even if it may display certain cognitive features. Moreover, even assuming that AI is intelligent, intelligence and cognition are not synonymous. In our opinion, one of the missing pieces in the design of a bodiless artificial intelligence would be the bodily characteristics and inputs, including from the gut-complex, which happen to be a crucial, causal, and constitutive component of cognitive capabilities. This might seem at odds with the liberal use of “cognition” we have adopted, but in fact the article shows a difference in the way of extending the term that remains consistent with our thesis.

^2^Although some authors have explained that they prefer the term “sensory experience” over that of “perception,” as the latter excludes illusory or hallucinatory episodes and suggest the intervention of higher mental faculties to transform the raw material of sensation into representations (Papineau, 2021). Here the term perception will be preferred. This is because, in contrast to sensation, perception can be more easily linked to the unconscious.

^3^One such possibility is to consider the body–brain relationship as a functional unit: e.g., some brain areas represent body structure and conditions. For instance, this is the case of the so-called Penfield homunculus (see Catani, 2017), which constitutes a map describing the density of sensory receptors or the number of cortical motor neurons present in that body area. In the case of perception, this means that the interaction of the body with the environment generates modifications in the structure and activities of the body that are conveyed (and therefore mapped) in specific brain areas. For example, a tactile sensation is transmitted to specific areas of the cortex that monitor the change in the sensory receptors. We can therefore speak genuinely of sensory representation. Similarly, in the most studied form of perception in cognitive science, i.e., sight/vision, it is possible to construct an account of this type. A certain type of bodily organization, equipped with specific sensory organs in certain positions, inevitably shapes the way in which the external stimulus is processed (in this sense, for example, an insect will inevitably have a different bodily starting point from ours). This also applies to a motor representation in the sense that the corporeal dimension of cognition allows a better grasp of the so-called peripersonal space, that is, the set of objects and processes of the surrounding environment which are close and in constant interaction with the body. The perceptual and cognitive field constituted by a body and its peripersonal space can therefore be understood as a functional and dynamic unit, which adapts as the environment changes (if new objects or constraints come into play) and of the body itself (since a biological body grows and modifies itself, either in its development or in its activities). Indeed, taking sight/vision as a paradigmatic model of perception (simplifying it a bit), we can recognize some aspects of the decentralization of the brain as the privileged seat of such activities. First of all, motor representation is determined by the representation of the body and not only on the object representation. Secondly, motor representation is intrinsically connected to particular forms of action. Another example of embodied cognition maintaining representational features relative to the body’s interior is the Embodied Interoceptive Coding model (EPIC) (Barrett and Simmons, 2015) that will be discussed further in this paper.

^4^Or, as in the case of the octopus in which the brain is present but its organization is very different from that of vertebrates, presenting instead a structure also distributed in the tentacles (which are therefore a constituent part of the “octopus brain”).
